# Use of the Abrysvo Vaccine in Pregnancy to Prevent Respiratory Syncytial Virus in Infants: A Review

**DOI:** 10.7759/cureus.68349

**Published:** 2024-08-31

**Authors:** Dhanvi Patel, Jyotsna Chawla, Cyril Blavo

**Affiliations:** 1 Department of Medicine, Nova Southeastern University Dr. Kiran C. Patel College of Osteopathic Medicine, Clearwater, USA; 2 Department of Foundational Sciences, Nova Southeastern University Dr. Kiran C. Patel College of Osteopathic Medicine, Clearwater, USA; 3 Department of Public Health and Pediatrics, Nova Southeastern University Dr. Kiran C. Patel College of Osteopathic Medicine, Clearwater, USA

**Keywords:** adverse effects, rsv vaccine, abrysvo, fda approved, pregnancy, infant health, maternal vaccination, respiratory syncytial virus

## Abstract

The FDA's approval of Pfizer’s new respiratory syncytial virus (RSV) prefusion (preF) vaccine, Abrysvo, marks a critical milestone in infant health and well-being by preventing lower respiratory tract infections in the most vulnerable. The vaccine has been approved for administration to pregnant women at 32 to 36 weeks of gestation and elderly people over 60. This review explores the Abrysvo vaccine, detailing its mechanism, efficacy, safety, and adverse events. It aims to inform healthcare providers about this vital method for safeguarding infant respiratory health through maternal immunization.

## Introduction and background

Respiratory syncytial virus (RSV) is an enveloped syncytial virus that poses a significant global health challenge as one of the most common causes of severe lower respiratory tract infections in children worldwide [1]. According to data collected by the CDC, over the 2023-2024 season, the overall rate of RSV-associated hospitalizations was 35.9 per 100,000, with most of these occurring in children between 0 and 4 years old in the United States [2]. RSV has been reported as the leading cause of childhood mortality and morbidity in children, with an estimated 97% of these RSV-associated deaths occurring in low- and middle-income countries [3].

Children under the age of five are more likely to be at higher risk of developing a serious lower respiratory infection from RSV because the developing airways in their lungs have a higher surface area to volume ratio [1]. Also, infants lack a robust immune system, making it easier for the virus to infect the infant and cause severe inflammation [4]. The risk is even higher for infants and children in developing countries, where higher reports of mortality have been documented when compared to the United States. Developing countries face limitations in resources and access to health care and often have ineffective government interventions that lead to an increased burden of the disease [5].

RSV-associated illness is also reported higher in the older adult population, with reports of 3-7% of healthy older adults being affected each year [6]. With age, the body experiences a decline in immune system function, weakening of respiratory muscles, and decreased levels of protective mucus and elastin in the lungs [7]. Due to these changes and the presence of other co-existing conditions, older adults tend to be at a higher risk for severe RSV-associated illness [6]. The burden of disease in older adults is high, with 177,000 hospitalizations and 14,000 deaths reported each year in the United States alone [6].

RSV is a single-stranded negative-sense genome subcategorized into two antigenic subgroups, RSV-A and RSV-B [3]. The RSV virus contains 11 proteins, with 8 of these proteins having a structural function [3]. One of these structural proteins is the fusion (F) protein responsible for initiating viral penetration and causing the fusion of the cellular and viral membranes [3]. The F protein's limited genetic diversity has rendered it an attractive target for antibody and vaccine therapies [8]. The F protein has two conformations, pre-fusion (pre-F) and post-fusion (post-F) [8]. The pre-F protein is the active form that serves as the target antigen for the development of vaccines; monoclonal antibodies to the pre-F protein are effective at neutralizing RSV [8].

In 2023, the FDA approved another RSV vaccine, Arexvy, a recombinant protein vaccine manufactured by GlaxoSmithKline (GSK) [9]. Arexvy comprises the prefusion RSV F antigen of the RSV-A strain and an adjuvant (AS01E) [9]. Arexvy has been approved for a single 0.5 mL intramuscular dose in adults 60 years of age and older [9]. Arexvy has not been approved for administration in pregnant women due to the increased risk of preterm births discovered during GSK’s phase 3 trials [10]. In 1998, the FDA approved Palivizumab, a monoclonal antibody to be administered to infants who are at high risk for severe RSV infection [8]. Palivizumab is administered monthly for up to five doses per patient and has resulted in a significant decrease in hospitalizations for high-risk infants [8]. However, the use of Palivizumab is restricted to only infants who are at high risk and do not meet the global need [11].

This review aims to comprehensively evaluate the safety, efficacy, and potential impact of the Abrysvo vaccine in preventing RSV in infants, with a particular emphasis on its use in pregnant females. Moreover, it seeks to address the gap in the existing literature by providing a synthesis of the latest evidence on maternal RSV vaccination, specifically focusing on findings from clinical trials and the Vaccine Adverse Event Reporting System (VAERS).

## Review

Abrysvo vaccine 

Abrysvo is a recombinant RSV vaccine manufactured by Pfizer and has been approved for administration in adults aged 60 years and older and pregnant women in 32 to 36 weeks of gestation [9]. Clinical trials were performed to assess the vaccine safety and efficacy for administration at the approved interval of 32 to 36 weeks of gestation, and the results stated that there is insufficient data to support or refute causation between the Abrysvo vaccine and preterm births [12]. The FDA approved administration in this gestation period as the benefits of RSV prevention in infants outweigh the risks associated with potential preterm birth adverse effects of the vaccine [12]. A comparison of the two vaccinations, Arexvy and Abrysvo, is shown in Table 1.

**Table 1:** Abrysvo and Arexvy vaccine comparison. *RSVPreF3 = RSV pre-Fusion 3 protein. **RSVPreF = RSV pre-Fusion protein.

Vaccine	Manufacturer	Indicated for	Type	Characteristics	Dosage	Method	Reference
Arexvy	GlaxoSmithKline (GSK)	Individuals >60 years	Recombinant protein vaccines	RSVPreF3*	Single dose 0.5 mL	Intramuscular injection	[12]
Abrysvo	Pfizer	Pregnant individuals of 32-36 weeks gestational age; individuals >60 years	Recombinant protein vaccines	RSVpreF**	Single dose 0.5 mL	Intramuscular injection	[13]

Mechanism of action of the Abrysvo vaccine 

**Figure 1:**
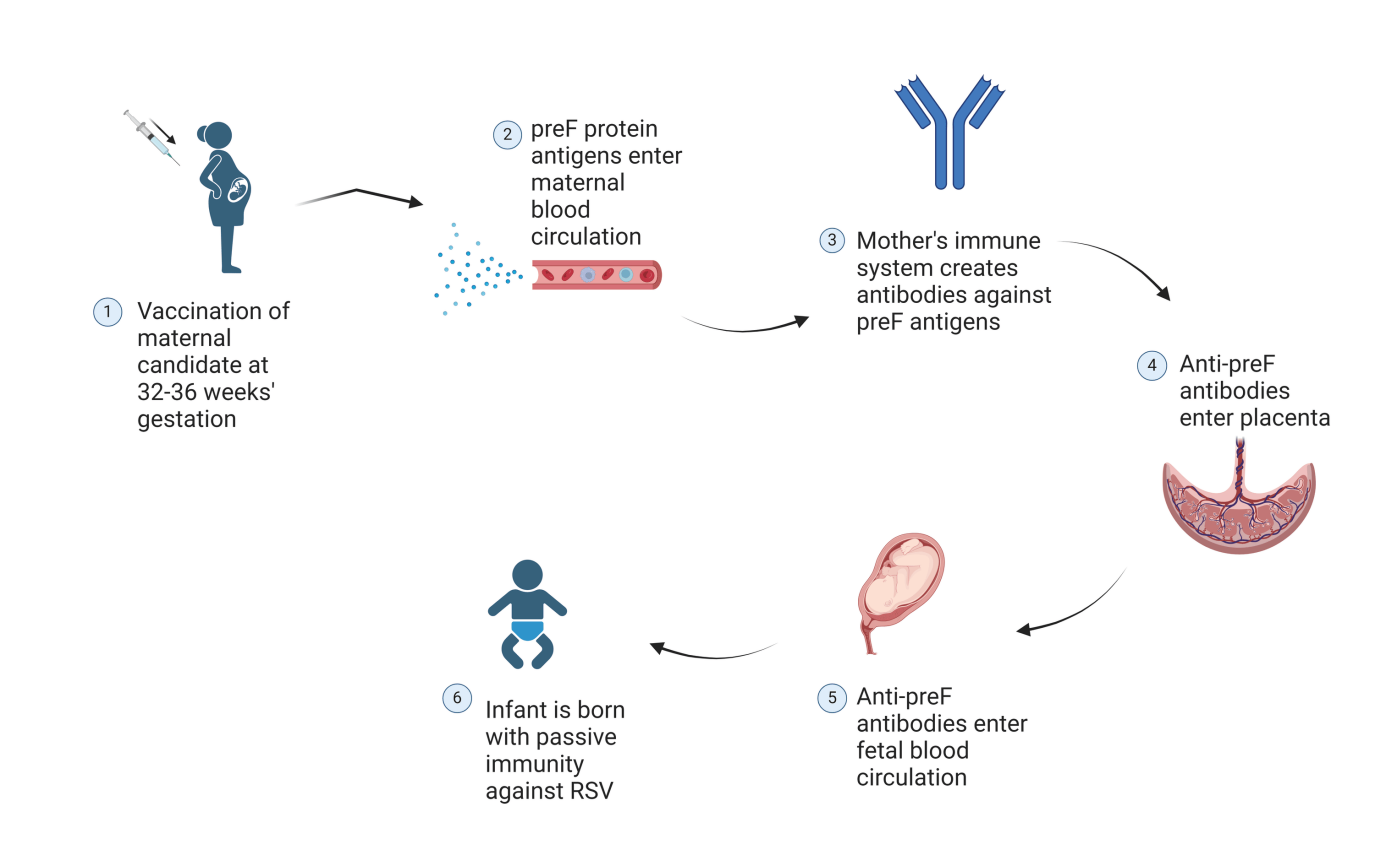
Abrysvo maternal antibody transfer. The process of passive immunization in infants at birth occurs through maternal vaccination, leading to antibody development in the mother subsequent to receiving the first FDA-approved RSV vaccine [16,17]. The illustration was created with BioRender.com. preF = pre-Fusion protein.

The Abrysvo vaccine is administered to the pregnant mother to protect the infant from RSV infection during the first six months of life [14,15]. Maternal immunization confers passive immunity to the fetus by transmitting antibodies developed in the mother post-vaccination across the placenta [16]. Passive transfer of antibodies is a highly effective method for initiating an immune response in infants who have yet to develop a fully functional immune system that is capable of combating infections like RSV. Maternal vaccines have been widely used, a common example being the Tetanus-Diphtheria-acellular Pertussis (Tdap) vaccine [15]. In addition to the transplacental transfer of antibodies, they can also confer protection to their infants through the transfer of antibodies via breastfeeding [8]. Abrysvo is characterized as a recombinant RSVpreF vaccine as it has been developed to contain antigens of the pre-fusion configuration of the F protein [11]. This vaccine mounts an immune response that creates neutralizing antibodies against these preF antigens administered, which will then prevent viral penetration and fusion of RSV into host cells [11]. The Abrysvo vaccine contains stabilized preF antigens against both antigenic subgroups, RSV-A and RSV-B, making it a bivalent vaccine [14]. Figure 1 depicts the mechanism of action of the Abrysvo vaccine.

Timeline and dosage 

**Figure 2:**
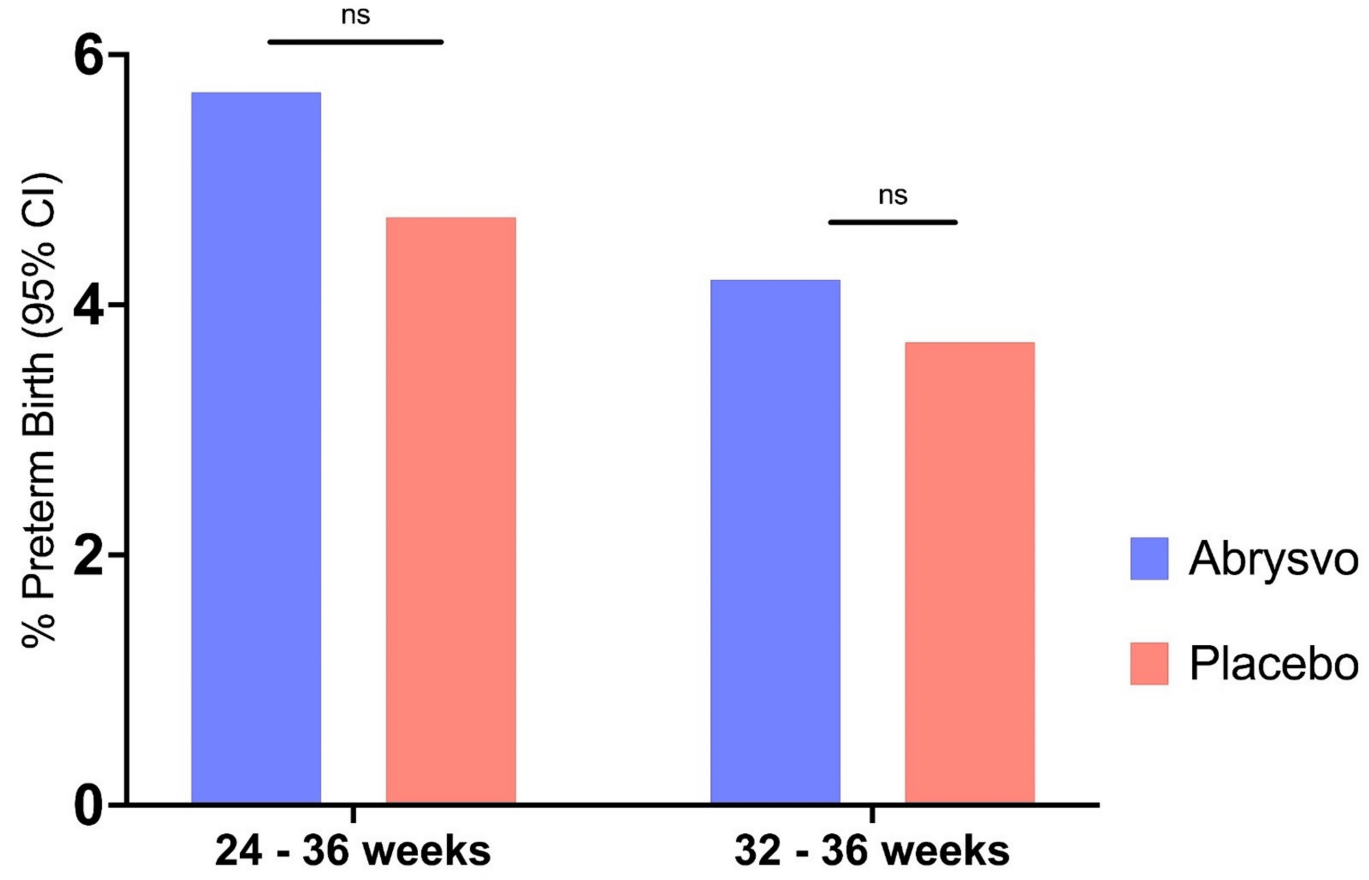
Preterm birth outcomes in Abrysvo vaccine phase 2b and 3 trial. Results of the phase 2b and 3 trials identifying increase in preterm births in Abrysvo administration during 24 to 36 weeks of gestation compared to the approved 32 to 36 weeks of gestation [14]. The figure was adapted from clinical trial data assessment results [14].

The Abrysvo vaccine has been approved by the FDA to be given at 32 to 36 weeks of pregnancy [12]. The FDA has approved Abrysvo to be administered as a single 0.5 mL dose given intramuscularly [12]. Clinical trials were performed using the Grading of Recommendations, Assessment, Development, and Evaluation (GRADE) approach to identify vaccine efficacy and safety when administered during the 32- to 36-week gestation period compared to the 24- to 36-week period [12]. These clinical trials collected data on the efficacy of vaccination against RSV-associated lower respiratory tract infection and adverse effects in pregnant females and infants [12]. Results obtained during this phase 3 trial revealed an increased preterm birth risk (less than 37 weeks gestation) with administration at 24 to 36 weeks of gestation when compared to 32 to 36 weeks of gestation [18]. Transplacental transfer of RSV-specific antibodies was greater when maternal vaccination was administered more than 30 days before birth, with evidence of 110-120% of maternal serum levels in infants compared to 60-80% in cases where maternal vaccination was administered less than 30 days before birth (Figure 2) [18]. The administration of this vaccine is contraindicated if the recipient is allergic to any of the components of Abrysvo and is likely to experience a severe response such as anaphylaxis [12].

Vaccine efficacy and safety 

A phase 3 randomized, double-blind clinical trial, MATISSE (Maternal Immunization Study for Safety and Efficacy), was conducted to test the vaccine safety and efficacy of the Abrysvo vaccine [17]. This clinical trial included pregnant women during their 24-36-week gestation period from 18 different countries [17]. The participants in this study were women under 49 years of age with uncomplicated and single pregnancies [17]. This study concluded that Abrysvo prevented medically attended severe RSV-associated lower respiratory tract illness in infants [17]. Within 90 days of birth, the vaccine showed 81.8% efficacy; within 180 days, it demonstrated 69.4% efficacy [17]. This clinical trial also observed safety endpoints in maternal participants and infants from birth through 12 months [17]. The study also concluded that there are no safety concerns for the mother and infants in their findings [17]. Mild-to-moderate immunogenicity was reported in mothers, along with serious adverse events similar to those reported in the placebo trials [17].

Adverse effects 

Adverse effects of a vaccine or drug indicate undesired harmful effects following the administration of that vaccine or drug [19]. The phase 3 MATISSE clinical trial was conducted to discover any potential adverse effects of the Abrysvo vaccine that would raise concerns about its approval, distribution, and administration [17]. This clinical trial aimed to examine adverse events in the maternal participants and the infants throughout their first 12 months of life. Key focal adverse events of interest in this study were preterm births, low birth weight, and developmental delay. The incidence of preterm births was reported to be 5.7% in the vaccine group and 4.7% in the placebo group [11]. The incidence of low birth weight was reported to be 5% in the vaccine group, while it was reported to be 4.1% in the placebo group. The incidence of developmental delay in the vaccine group and placebo group were both reported to be 0.3% each. 

This clinical trial also reported other adverse events in their findings [17]. These adverse events were predominantly disorders associated with pregnancy, puerperium, and perinatal conditions [11]. The most prevalent conditions observed were pre-eclampsia, characterized by high blood pressure in the pregnant mother, and the inherent risks of fetal distress syndrome secondary to hypoxia in the fetus [17].

Another adverse effect reported in the first months of life was neonatal jaundice, with an incidence of 7.2% in the vaccine group and 6.7% in the placebo group [11]. Adverse effects, such as eczema, asthma, and gastroesophageal disease, were similar between the placebo and vaccine groups [11]. As these incidences of adverse events were not significantly different from the outcomes reported in the placebo group, the FDA approved the Abrysvo vaccine [11]. In conclusion, the overall benefits of preventing RSV-associated lower respiratory tract infection by this vaccination surpass the potential adverse effects of the vaccine [12]. Table 2 highlights the adverse events in Abrysvo versus placebo groups.

**Table 2:** Adverse events in Abrysvo versus placebo.

Trial	Abrysvo	Placebo	Reference
Pre-eclampsia	1.8%	1.4%	[13]
Fetal distress syndrome	1.8%	1.4%	[13]
Preterm births	5.7%	4.7%	[17]
Fetal death	0.3%	0.2%	[13]

VAERS insights on adverse events

The VAERS is managed by the CDC and FDA and is designed to monitor and evaluate the safety of vaccines through passive public reporting [20]. To evaluate their consistency, we compared the adverse events reported in the clinical trial with those documented by healthcare professionals and users in the VAERS database [21].

According to data retrieved from the VAERS online database on April 12, 2024, a total of 199 serious post-vaccination symptoms were reported between January 1, 2023, and April 12, 2024, affecting infants and adults from the ages of 0 to 39 years [21]. The most reported adverse event was premature labor or delivery, with 28 reports in total. Of these 28 reports, 21 were reported by females between the ages of 30 and 39. The second most reported adverse event was cesarean section deliveries, with eight reports in total. The third most reported adverse event was preterm premature rupture of membranes, with six reports in total.

The adverse events commonly seen in the VAERS data correlate with the findings of the clinical trials. Premature births reported most frequently in the VAERS data, had an incidence of 5.7% in the MATISSE clinical trial [17]. However, the MATISSE trial did not report cesarean section deliveries, which were observed in the VAERS data, necessitating further investigation to confirm or deny a causal link with the Abrysvo vaccine [17]. It is important to note that while the VAERS data supports the adverse effects discovered in the phase 3 clinical trial, it is not sufficient to establish a cause-and-effect relationship. VAERS reports are collected passively and may contain incorrect, biased, and either under- or over-reported information. Due to its limitations, including potential reporting biases, the VAERS data requires careful interpretation. Continued monitoring and comprehensive reviews are essential to accurately assess the Abrysvo vaccine's safety.

## Conclusions

Pfizer’s RSV preF maternal vaccine, marketed as Abrysvo, represents a significant milestone in reducing infant hospitalizations and medically attended respiratory tract infections. The FDA has approved Abrysvo for administration between 32 and 36 weeks of pregnancy, deeming it safe and efficacious based on promising clinical trial results. However, further studies are needed to evaluate potential adverse effects. Independent research on preterm births, pre-eclampsia, and fetal distress syndrome using large, representative sample sizes can provide more comprehensive safety data and reduce vaccine hesitancy. Educating clinicians about Abrysvo's potential and encouraging informed discussions with expectant mothers will be crucial in maximizing the vaccine's benefits. This proactive approach can significantly enhance infant respiratory health and mitigate the global burden of RSV.
